# Combined Minimally Invasive Mitral Valve Surgery and Percutaneous Coronary Intervention: A Hybrid Concept for Patients with Mitral Valve and Coronary Pathologies

**DOI:** 10.3390/jcm12175553

**Published:** 2023-08-26

**Authors:** Martín Moscoso-Ludueña, Maximilian Vondran, Marc Irqsusi, Holger Nef, Ardawan J. Rastan, Tamer Ghazy

**Affiliations:** 1 Department of Cardiac and Vascular Surgery, Rotenburg Heart and Vascular Centre, 36199 Rotenburg an der Fulda, Germany; m.moscoso-luduena@hkz-rotenburg.de (M.M.-L.);; 2 Department of Cardiac and Vascular Surgery, Klinikum Karlsburg, Heart and Diabetes Center Mecklenburg-Western Pommerania, 17495 Carlsburg, Germany; vondran.m@drguth.de; 3 Department of Cardiac Surgery, Marburg University Hospital, Baldingerstrasse, 35043 Marburg, Germany; irqsusi@med.uni-marburg.de; 4 Department of Cardiology, Rotenburg Heart and Vascular Centre, 36199 Rotenburg an der Fulda, Germany; 5 Department of Cardiology, Giessen University Hospital, 35392 Giessen, Germany

**Keywords:** mitral valve, minimally invasive, PCI, hybrid

## Abstract

We evaluated the feasibility of hybrid percutaneous coronary intervention (PCI) and minimally invasive mitral valve surgery (MIMVS) in patients with concomitant coronary and mitral disease. Of 534 patients who underwent MIMVS at our institution between 2012 and 2018, those with combined mitral and single vessel coronary pathologies who underwent MIMVS and PCI were included. Patients were excluded if they had endocarditis or required emergency procedures. Preprocedural, procedural, and postprocedural data were retrospectively analyzed. In total, 10 patients (median age, 75 years; 7 males) with a median ejection fraction (EF) of 60% were included. Nine patients underwent PCI before and one after MIMVS. The success rate was 100% in both procedures. There were no postoperative myocardial infarctions or strokes. Two patients developed delirium and one required re-thoracotomy for bleeding. The median stay in intensive care and the hospital was 3 and 8 days, respectively. The 30-day survival rate was 100%. A hybrid PCI and MIMVS approach is feasible in patients with mitral valve and single vessel coronary disease. In combined pathologies, the revascularization strategy should be evaluated independent from the mitral valve pathology in the presence of MIMVS expertise. Extension of this recommendation to multivessel disease should be evaluated in future studies.

## 1. Introduction

There is an increasing demand for minimally invasive mitral valve surgery (MIMVS) because of the cosmetic benefits, shorter hospital stay, and avoidance of sternotomy and its wound complications, making it a more desirable option than sternotomy. The aim of MIMVS is to treat patients with mitral valve pathologies using a favorable surgical approach [1,2]. However, the presence of a concomitant coronary pathology might influence the treatment strategy for both the coronary pathology and the mitral valve disease. According to the European Society of Cardiology (ESC) and the European Association of Cardiothoracic Surgery (EACTS) guidelines for myocardial revascularization, the choice of revascularization strategy (percutaneous coronary intervention [PCI] vs. coronary artery bypass grafting [CABG]) might be influenced by the presence of any indications of further cardiac procedures, such as valve surgery [3]. Based on these recommendations, patients with concomitant mitral valve and coronary pathologies—who are candidates for concomitant surgical revascularization according to the latest ESC/EACTS guidelines—are usually operated on via the median sternotomy to allow sufficient exposure of the coronary arteries [3].

The guidelines do not differentiate between single, double, or triple vessel disease in cases of concomitant valve surgery or between minimally invasive or classic approaches for valve therapy. Single vessel disease, which can be treated sufficiently endovascularly, is recommended to be treated surgically if concomitant valve surgery is to be performed [3]. Yet, this decision might be the leading indication for changing the surgical approach and denying the patient a minimally invasive procedure. The feasibility of MIMVS and PCI as a hybrid concept for patients with mitral valve and coronary pathologies is yet to be investigated. The aim of this study was to present our institution’s initial experience with this hybrid concept in patients with combined pathologies.

## 2. Materials and Methods

### 2.1. Patient Cohort

Between January 2012 and April 2018, 534 patients who underwent isolated or combined MIMVS at our institution were screened for inclusion in this retrospective descriptive study. Patients were included if they had a combined mitral pathology and an indication for single vessel coronary revascularization and underwent hybrid therapy with MIMVS and interventional therapy for coronary disease. Patients who underwent a non-planned PCI or emergency procedures and those with endocarditis were excluded from the study. The study was approved by the institutional review board.

### 2.2. Data Collection

All data were collected from the patient records. The pre-management patient data, decision of the heart team, PCI, and operative procedural details as well as post-management results were retrospectively collected and analyzed.

### 2.3. Statistical Analysis and Reporting

Continuous variables are presented as medians with interquartile ranges (IQRs). Categorical variables are presented as absolute numbers. All statistical tests were performed using SPSS v22.0 (IBM Inc., Armonk, NY, USA).

## 3. Results

Overall, 10 patients who met the inclusion criteria were included in the analysis.

### 3.1. Demographic and Medical Data

Table 1 and Table 2 summarizes the patient demographic and medical data. The overall median age was 75 years and seven patients were males. The median body mass index was 29.4 (23.7–31.6) kg/m^2^. The comorbidities included hyperlipidemia (*n* = 7), renal insufficiency (*n* = 4), and diabetes mellitus (*n* = 3). The median creatinine value in the patient cohort was 1.08 mg/dl (0.84–1.87). None of the patients were on dialysis or had chronic obstructive pulmonary disease (COPD), liver failure, or peripheral vascular disease.

The analysis of the cardiac data revealed a median left ventricular ejection fraction (EF) of 60% (40–60%). Three patients had atrial fibrillation. All patients were of New York Heart Association (NYHA) class 3. Two patients were of Canadian Cardiac Society (CCS) class 2. All patients had mitral valve insufficiency with no stenosis and one patient had tricuspid regurgitation. None of the patients had aortic valve or aortic diseases. There were no cases of acute myocardial infarctions. Four patients had a past history of myocardial infarction; one patient each had it within 1 month and 90 days before the procedure, while two patients had it more than 90 days before the procedure. Two patients had a history of CABG in 1993 and 2003, respectively, both of whom underwent totally arterial bypass grafting to the left coronary system with no bypass grafting to the then non-diseased right coronary system that required intervention in the present study. Coronary angiography confirmed patent bypass grafts. None of the patients had a previous mitral valve surgery. The details of coronary findings are summarized in Table 1 and those of the mitral valve pathology are summarized in Table 2.

### 3.2. Procedural Data

#### 3.2.1. Coronary Intervention

Nine patients underwent PCI with stent implantation before the surgery. The median time between PCI and MIMVS was 48 (IQR, 8–63) days. One patient who had previously undergone cardiac surgery underwent the current coronary intervention 48 days after the procedure. Overall, the procedural success rate was 100%. The stents used included Coroflex, Coroflex Blue, Coroflex Blue Ultra, Coroflex ISAR (B. Braun Melsungen AG, Melsungen, Germany), and Xience pro (Abbott Vascular, Santa Clara, CA, USA). There were no periprocedural complications. All patients received dual antiplatelet therapy with acetylsalicylic acid and clopidogrel. Table 3 summarizes the details of the coronary interventions performed.

#### 3.2.2. Surgical Procedure

MIMVS was performed as previously described [4]. In brief, the patient was brought to the operation theatre, placed under general anesthesia, and intubated with a single-lumen endotracheal tube. The patient was then put on cardiopulmonary bypass (CPB) via femoral arterial and venous cannulae. In case of concomitant tricuspid valve surgery, a second venous cannula was inserted into the right jugular vein. After dissecting pleural adhesions, if present, via an anterolateral minithoracotomy, the CPB was initiated and the heart and aorta were exposed. In eight patients, the aorta was cross-clamped with a Valve Gate™ transthoracic aortic clamp (Geister, Tuttlingen, Germany) and antegrade Bretschneider’s Custodiol^®^ crystalloid cardioplegia (Dr. Franz Köhler Chemie, Bensheim, Germany) was administered via the aortic root, followed by left atriotomy and exposure of the mitral valve with a Valve Gate™ special minimally invasive atrial retractor (Geister, Tuttlingen, Germany). In one patient with extensive intrapericardial adhesions and in one of two patients who had previously undergone coronary surgery, cross-clamping of the aorta was not possible due to excessive adhesions; therefore, atriotomy and mitral valve exposure were performed in a beating heart. In these two patients, the heart was put into ventricular fibrillation directly before introducing the mitral ring into the left atrium to prevent an air embolism after achieving a competent valve. After completion of the mitral valve repair, the left atrium was closed and the aortic clamp was removed (or the heart defibrillated in the two aforementioned cases). This was followed by gradual weaning from the cardiopulmonary bypass and wound closure after echocardiographic confirmation of successful valve repair. Table 4 summarizes the surgical details of all the patients.

The median operative time was 230 (IQR, 200–255) minutes. The median CPB time was 144 (IQR, 124–152) minutes. The median ischemic time in patients who underwent aortic cross-clamping was 80 (IQR, 66–89) minutes. All valves were successfully repaired. There were no cases of valve replacement. There was no conversion to sternotomy and no intraoperative complications.

### 3.3. Postoperative Data Analysis

Table 5 summarizes the postoperative results. There was no case of postoperative low cardiac output requiring inotropic support or mechanical support or of myocardial infarction or postoperative stroke. Two patients developed postoperative delirium. One patient underwent re-thoracotomy for bleeding. Blood transfusion was needed in one patient perioperatively and in five patients during the postoperative period. One of the three patients who underwent ablation regained sinus rhythm, while the other two demonstrated persistent atrial fibrillation. No cases required permanent pacemaker implantation. One patient with postoperative sepsis was successfully treated with antibiotics with no further complications. The median ventilation time was 20 (IQR, 14–24) hours. The median duration of stay in intensive care was 3 (IQR, 2–4) days and the median duration of hospital stay was 8 (IQR, 8–11) days. There was no case of in-hospital mortality. The 30-day survival rate was 100%.

## 4. Discussion

Prospective randomized trials did not demonstrate a survival benefit of MIMVS over traditional surgery via sternotomy [1,2,5]. However, previous studies did report better cosmetic results and shorter hospital stays with MIMVS over traditional surgery via sternotomy [1,2]. As both factors are welcomed by both patients and clinicians, an increasing number of patients with mitral valve pathologies are undergoing the procedure via the minimally invasive approach [6,7,8,9]; over 50% of those who undergo isolated mitral valve surgery in Germany do so via the minimally invasive approach [9].

Although the recent guidelines on valvular heart disease recommend the CABG procedure in the presence of coronary heart disease (CHD) in patients with primary indications for aortic or mitral valve surgery, they recommend PCI in patients who undergo catheter-based intervention of either valve [3,10]. These recommendations are understandable because the CABG procedure is favorable in patients who undergo sternotomy for the valve surgery and PCI is favorable in those who receive treatment percutaneously. Although previous reports have been published reporting favorable results of a hybrid concept consisting of PCI and MIMVS in different patient cohorts with mitral valve and coronary disease [11,12,13,14], there are no recommendations regarding the revascularization strategy in patients who might undergo MIMVS in the presence of adequate expertise.

**Figure 1:**
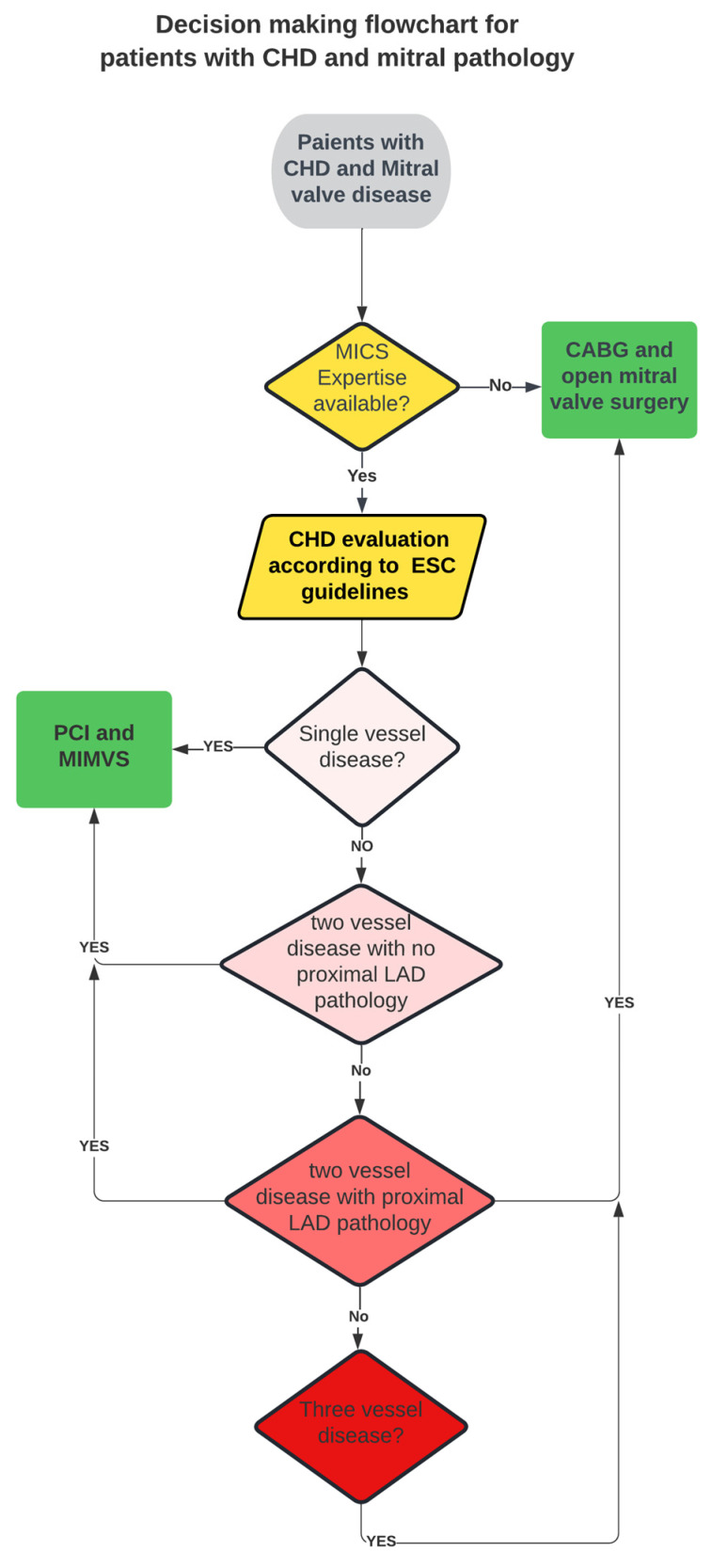
**Decision-making flowchart for patient selection**. Abbreviations: CHD: coronary heart disease; MICS: minimally invasive cardiac surgery; CABG: coronary artery bypass grafting; ESC: European Society of Cardiology; LAD: left anterior descending artery; PCI: percutaneous coronary intervention; MIMVS: minimally invasive mitral valve surgery.

In this study, we evaluated the decision-making process and results of this hybrid concept. The strategy of the heart team was to analyze CHD independent from the mitral valve pathology. If CHD was to be treated endovascularly according to the recent guidelines with no mitral valve pathology, this hybrid concept was suggested [3]. The rationale behind this concept was to not deny the patient mitral valve repair via a favorable approach due to coronary pathology that would have been otherwise treated endovascularly with expected good results. Additionally, the rationale is also to facilitate the approach to the mitral valve in re-do procedures after a previous CABG with patent grafts where avoiding re-sternotomy is of special benefit to avoid graft injury. The results of this study demonstrate that this concept is a viable option in the presence of adequate expertise in MIMVS. Based on this study, we developed a simple decision-making flowchart to help in patient selection (Figure 1).

Another important aspect is the sequence of the procedures. On one hand, it might be surgically favorable to perform the surgical procedure before initiating double antiplatelet therapy (DPAT) after endovascular therapy for CHD because randomized controlled trials and retrospective studies have demonstrated higher rates of postoperative bleeding in patients on DPAT [15,16,17,18,19]. On the other hand, it is crucial to ensure adequate coronary perfusion before the surgery or at least ensure adequate myocardial protection with cardioplegia and avoid periprocedural myocardial infarction because previous publications have demonstrated a higher rate of perioperative myocardial infarctions in patients whose diseased coronary vessels were not addressed [20,21]. In our study cohort, we generally opted to perform PCI first to also confirm the success of myocardial revascularization before MIMVS.

Regarding the optimum timeframe between PCI and MIMVS, the heart team unfortunately did not reach a consensus at the time of performing the procedures. Post-PCI clopidogrel therapy was stopped 5 days before the surgery in patients who were operated on after more than 30 days of PCI according to the ESC/EACTS guidelines [22]. In patients with severe valvular symptoms who were operated on within 1 month of PCI, surgery was performed under DAPT. Our analysis did not demonstrate higher bleeding tendency in these patients. Furthermore, the analysis did not reveal periprocedural myocardial infarctions or low cardiac output syndrome, which reflect adequate myocardial perfusion and protection. Therefore, we believe that it is advisable to first address the CHD to ensure successful revascularization before surgery and to optimize myocardial protection during surgery.

The patients who had previously undergone CABG and were to undergo re-do surgery represented a special cohort. In the first patient, the heart team preferred to perform the mitral valve surgery first to avoid the bleeding tendencies in re-do situations. As the surgical team did not face increased bleeding tendency in this patient, the second patient underwent PCI first and then surgery, which was performed without complications. In hindsight, while believing that PCI should precede MIMVS, we also believe that it is advisable to plan PCI well before MIMVS to be able to safely stop DAPT before the surgery to decrease the bleeding risk without highly increasing the risk of stent thrombosis.

This study has several limitations. It was a single center study that included a small number of patients, and procedures were not randomly assigned. To our knowledge, there are no previous studies that have evaluated the feasibility of hybrid PCI and MIMVS in similar cohorts. Therefore, further studies with larger patient numbers are required to corroborate the results of the present study and to draw more robust conclusions. Furthermore, the patient management protocol did not adhere to a standardized protocol regarding the timeframe between the procedures. Further studies should adhere to a standardized timeframe to provide clearer recommendations. Nevertheless, MIMVS might be more tolerant of DAPT with less restrictive indications for surgery under DAPT. This is yet to be confirmed in further studies.

## 5. Conclusions

A hybrid concept of PCI and MIMVS is feasible in patients with mitral valve pathology and single vessel coronary disease and can be considered if the team members have sufficient expertise in MIMVS. Future studies should also evaluate whether this recommendation is feasible for patients with multivessel disease, so that for patients with combined mitral valve and coronary pathologies, with the availability of adequate expertise in MIMVS, the choice of revascularization strategy can be evaluated independent from the mitral valve pathology.

Based on the results of this report, in centers with expertise in MIMVS, the coronary pathology and the choice revascularization strategy should be made independent from the mitral valve pathology. In patients whose coronary pathology is suitable for PCI, a hybrid PCI and MIMVS should be considered.

## Figures and Tables

**Table 1:** Preoperative data.

Variables	Result
*Demographic data*	
Age, years (IQR)	75 (64–81)
Males, *n*	7
Body mass index, kg/m^2^ (IQR)	29.4 (23.7–31.6)
*Risk factors*	
Arterial hypertension, *n*	9
Diabetes mellitus, *n*	3
Hyperlipidemia, *n*	7
History of smoking, *n*	1
Renal insufficiency, *n*	4
Previous cardiac surgery, *n*	2
EuroSCORE II, % (IQR)	8.1 (2.5–8.6)
STS score, % (IQR)	1.2 (0.7–3.1)
*Cardiac data*	
NYHA class, median	3
Mitral valve insufficiency, *n*	10
Ejection fraction, % (IQR)	60 (40–60)
Tricuspid valve insufficiency, *n*	1
Pulmonary hypertension >60 mmHg, *n*	0
History of myocardial infarction, *n*	4
Atrial fibrillation, *n*	3
Pacemaker/AICD, *n*	1
*Coronary data*	
* Previously operated patients*	
Patient No.1 CABG	LIMA to D1 and LAD, RIMA-T to M1, intact grafts
Patient No.2 CABG	LIMA to LAD, RIMA-T to M1, intact grafts
RCA as Target vessel for PCI	both patients
* Previously non-operated patients*	
RCA as Target vessel for PCI, n	1
LAD as Target vessel for PCI, n	3
D1 as Target vessel for PCI, n	1
RCX as Target vessel for PCI, n	3

**Abbreviations:** NYHA: New York Heart Association; AICD: automated implantable cardioverter defibrillator; CABG: coronary artery bypass grafting; IQR: interquartile range; LIMA: left internal mammary artery; D1: the first diagonal branch; LAD: the left anterior descending artery; RIMA-T: the right internal mammary artery as a t-graft from the left internal mammary artery; M1: the first marginal branch of the circumflex artery; MIDCAB: minimally invasive direct coronary artery bypass; RCA: right coronary artery; RCX: circumflex artery.

**Table 2:** Preoperative EF and mitral valve pathology.

Patient No.	Preop. EF	Mitral Valve Pathology
1	60%	Mixed regurgitation due to atrial remodeling due to - Long persistent atrial fibrillation (Carpentier type I) and - A2 prolapse due to chordal elongation
2	60%	Mixed regurgitation due to - atrial remodeling due to paroxysmal atrial fibrillation (Carpentier type I) and - local A1 prolapse due to chordal elongation
3	50%	Primary regurgitation due to P3 flail due to P3 chordal rupture
4	37%	Secondary regurgitation due to PML restriction due to LV remodeling and ICM
5	40%	Secondary regurgitation due to PML restriction due to LV remodeling and ICM
6	59%	Secondary regurgitation due to atrial remodeling due to long persistent atrial fibrillation (Carpentier type I)
7	30%	Secondary regurgitation due to AML restriction due to LV remodeling and ICM
8	60	Primary regurgitation due to P2 flail due to P2 chordal rupture
9	60	Primary regurgitation due to P2 flail due to P2 chordal rupture
10	60	Primary regurgitation due to Barlow disease with bileaflet billowing and pronounced P2 prolapse due to chordal elongation

**Abbreviations:** AML: Anterior Mitral Leaflet; EF: left ventricular ejection fraction; A1, A2, P2, P3: anterior and posterior segments of the mitral leaflets; LV: left ventricle; PML: posterior mitral leaflet; ICM: ischemic cardiomyopathy.

**Table 3:** Procedural data.

Patient No.	PCI before or after Surgery	Time Span (Days)	Target Vessel	Number of Stents	Stent Type
1	Before	17	LAD Segment 6	1	80% proximal to DES → BMS (coroflex Blue 3.0/14 proximal to DES)
2	Before	8	RCA Segment 3	1	BMS (Coroflex Blue Ultra 2.5/14)
3	Before	21	D1 Segment 9	1	DES Coroflex ISAER 2.5/14
4	Before	63	RCA multiple segments	2	Segment 1: POBA 4.0 Balloon 10 bar due to previous stent stenosis Segment 2: DES Coroflex ISAR 4.0/15 Segment 3 DES Coroflex ISAR 2.75/19
5	Before	66	RCX segment 12	1	DES (Xience pro 2.75/12)
6	Before	62	RCX segment 13	1	DES (Coroflex ISAR 3.0/19)
7	Before	61	LAD segment 6	1	DES (Coroflex ISAR 3.0/14)
9	After	48	RCA segment 2	1	BMS (Coroflex Blue 4.0/25)
10	Before	7	RCX segment 13	2	2x BMS (2x Coroflex 2.5/9)
11	Before	3	LAD segment 7	1	BMS (Coroflex 3.0/25)

**Abbreviations:** PCI: percutaneous coronary angioplasty; LAD: left anterior descending artery; RCA: right coronary artery; D1: the first diagonal branch of LAD; RCX: circumflex artery; DES: drug eluting stent; BMS: bare metal stent; POBA: plain old balloon angioplasty.

**Table 4:** Surgical data.

Patient No	Redo Surgery	Beating Heart Surgery	Surgery on DAPT	Surgery	Repair Technique
1	No	No	No	MV repair	32 mm Ring, A2–A3 plication
2	No	No	Yes	MV repair, Cryoablation	32 mm Ring, A1–P1 edge-to-edge stitch, P1–P2 indentation closure
3	No	No	Yes	MV repair, Cryoablation	32 mm Ring, P2 triangular resection
4	Yes	Yes	Yes	MV repair	36 mm Ring
5	No	Yes	Yes	MV repair	30 mm Ring
6	No	No	Yes	MV and TV repair, Cryoablation	28 mm Ring, 34 mm tricuspid band
7	No	No	Yes	MV repair	32 mm Ring
9	Yes	No	No	MV repair	30 mm Ring, P2 triangular resection
10	No	No	Yes	MV repair, PFO–closure	34 mm Ring, P2 triangular resection
11	No	No	Yes	MV repair	32 mm Ring, P2 triangular resection

**Abbreviations:** DAPT: double antiplatelet therapy; MV: mitral valve; TV: tricuspid valve; PFO: patent foramen ovale.

**Table 5:** Postoperative results.

Variable	Result
Postoperative myocardial infarction	0
Postoperative stroke	0
Postoperative delirium, *n*	2
Drain volume, mL (IQR)	1100 (850–1450)
Re-thoracotomy for bleeding, *n*	1
Perioperative blood transfusion, *n*	1
Postoperative blood transfusion, *n*	5
Ventilation time, hours (IQR)	10 (14–24)
ICU time, days (IQR)	3 (2–4)
Hospital stay, days (IQR)	8 (8–11)
30-day survival rate, %	100

**Abbreviations:** IQR: interquartile range.

## Data Availability

The data presented in this study are available on request from the corresponding author. The data are not publicly available due to data privacy regulation of the ethical committee.
